# Predictive determinants of overall survival among re-infected COVID-19 patients using the elastic-net regularized Cox proportional hazards model: a machine-learning algorithm

**DOI:** 10.1186/s12889-021-12383-3

**Published:** 2022-01-05

**Authors:** Vahid Ebrahimi, Mehrdad Sharifi, Razieh Sadat Mousavi-Roknabadi, Robab Sadegh, Mohammad Hossein Khademian, Mohsen Moghadami, Afsaneh Dehbozorgi

**Affiliations:** 1 Department of Biostatistics, School of Medicine, Shiraz University of Medical Sciences, Shiraz, Iran; 2 Emergency Medicine Research Center, Shiraz University of Medical Sciences, Shiraz, Iran; 3 Emergency Medicine Department, School of Medicine, Shiraz University of Medical Sciences, Shiraz, Iran; 4 Department of Medical Surgical Nursing, School of Nursing and Midwifery, Shiraz University of Medical Sciences, Shiraz, Iran; 5 Noncommunicable Disease Research Center, Shiraz University of Medical Sciences, Shiraz, Iran

**Keywords:** COVID-19, Elastic-net, Machine-learning, Re-infection, Survival

## Abstract

**Background:**

Narrowing a large set of features to a smaller one can improve our understanding of the main risk factors for in-hospital mortality in patients with COVID-19. This study aimed to derive a parsimonious model for predicting overall survival (OS) among re-infected COVID-19 patients using machine-learning algorithms.

**Methods:**

The retrospective data of 283 re-infected COVID-19 patients admitted to twenty-six medical centers (affiliated with Shiraz University of Medical Sciences) from 10 June to 26 December 2020 were reviewed and analyzed. An elastic-net regularized Cox proportional hazards (PH) regression and model approximation via backward elimination were utilized to optimize a predictive model of time to in-hospital death. The model was further reduced to its core features to maximize simplicity and generalizability.

**Results:**

The empirical in-hospital mortality rate among the re-infected COVID-19 patients was 9.5%*.* In addition, the mortality rate among the intubated patients was 83.5%. Using the Kaplan-Meier approach, the OS (95% CI) rates for days 7, 14, and 21 were 87.5% (81.6-91.6%), 78.3% (65.0-87.0%), and 52.2% (20.3-76.7%), respectively. The elastic-net Cox PH regression retained 8 out of 35 candidate features of death. Transfer by Emergency Medical Services (EMS) (HR=3.90, 95% CI: 1.63-9.48), SpO_2_≤85% (HR=8.10, 95% CI: 2.97-22.00), increased serum creatinine (HR=1.85, 95% CI: 1.48-2.30), and increased white blood cells (WBC) count (HR=1.10, 95% CI: 1.03-1.15) were associated with higher in-hospital mortality rates in the re-infected COVID-19 patients.

**Conclusion:**

The results of the machine-learning analysis demonstrated that transfer by EMS, profound hypoxemia (SpO_2_≤85%), increased serum creatinine (more than 1.6 mg/dL), and increased WBC count (more than 8.5 (×10^9^ cells/L)) reduced the OS of the re-infected COVID-19 patients. We recommend that future machine-learning studies should further investigate these relationships and the associated factors in these patients for a better prediction of OS.

**Supplementary Information:**

The online version contains supplementary material available at 10.1186/s12889-021-12383-3.

## Background

Severe acute respiratory syndrome coronavirus 2 (SARS-CoV-2) disease 2019 (COVID-19) first started in China in December 2019. It rapidly spread around the world and became a pandemic and a major health issue. It is associated with clinical symptoms [1, 2]. The World Health Organization (WHO) stated that infected patients can be considered as non- infectious after complete symptomatic recovery and two negative real-time reverse transcription polymerase chain reaction (RT-PCR) tests [3]. The median time from symptom onset to the detection of immunoglobulin (Ig) M antibody was reported twelve days and was determined as fourteen days for IgG antibodies. However, it is not clear how long the protection will last [4].

Recently, global concern over the possibility of re-infection with SARS-CoV-2 has risen considerably [5, 6]. Studies from different parts of the world have reported that some patients (especially those with underlying diseases) treated and recovered from COVID-19 may have new symptoms with COVID-19 re-infection. The COVID-19 re-infection can be confirmed through epidemiological, clinical, radiological, serological, and genomic studies [5, 7–10]. It is worth mentioning that re-infection is possible in the other members of the coronavirus family. Therefore, immunity to COVID-19 is not persistent and containing the virus will be difficult [11].

So far, various studies have been conducted to explore the determinants of the overall survival (OS) of COVID-19 patients [12–17]. Epidemiological studies have shown that several factors affect the OS of COVID-19 patients including gender, age, cardiovascular diseases, D-dimer, white blood cells (WBC) count, intensive care unit (ICU) admission, chronic kidney disease, hospitalization, neutrophil-to-lymphocyte ratio (NLR), and intubation [12, 14, 15, 18].

In the study of time-to-event data (e.g. time to death or discharge), bigger sample sizes and more desired events are often preferable. Simulation studies have indicated that training multiple survival time models using traditional models with small sample size data can lead to bias in the estimation of the coefficients since the outcome events per candidate feature (OEPCF) are too few. Accordingly, the model will most probably have unstable predictions and a poor performance on new datasets [19–21]. Among the different methods to model the survival data, the Cox proportional hazards (PH) model is the most popular approach because it has fewer assumptions than parametric models [22, 23]. Based on the rule of thumb, a minimum of between five and twenty OEPCF is needed for reliable results in the Cox-adjusted PH regression model [19–21]. For small sample size data, if the number of the candidate features is relatively large, the number of the OEPCF tends to be less than expected and using traditional survival models can be misleading [20, 21]. In such cases, using least absolute shrinkage and selection operator (LASSO) and elastic-net regularized Cox PH models through machine-learning (ML) algorithms is the better option [21, 24].

Generally, narrowing a large set of features to a smaller one can improve our understanding of the most important risk factors for in-hospital death in patients with COVID-19. The LASSO and *elastic-net can be applied to a dataset to produce estimates of regression coefficients via* adding a penalty term to the partial log-likelihood function*. *When combined with ML algorithms for feature selection we can get an externally validated parsimonious regression model [24].

To the best of our knowledge, the prognosis and OS of patients with COVID-19 re-infection have not been determined so far. Hence, the current study aimed to derive a parsimonious regression model for predicting OS among re-infected COVID-19 patients. In this study, the elastic net ML algorithm (which has not been utilized for COVID-19 data so far) was used to optimize the prediction of time to in-hospital death.

## Methods

### Design and study population

This retrospective cohort study was conducted on all inpatients with confirmed COVID-19 who were referred to 26 medical centers (affiliated with Shiraz University of Medical Sciences (SUMS)) from 10 June to 26 December 2020. Their disease was confirmed by RT-PCR test. The inclusion criteria were patients with the age of ≥18 years who had previously recovered from COVID-19 disease but were re-infected. Patients with unknown last status (in-hospital death or discharge from the hospital) and high missing data were excluded from the study. Finally, a total of 283 cases were analyzed. The patients’ demographics characteristics and clinical and laboratory test findings available soon after admission to the hospital were extracted from the Health Information System (HIS) of SUMS.

This study was conducted in accordance with the Declaration of Helsinki. Besides, it was approved by the Vice-Chancellor of Research and Technology (Grant No. 21237) as well as the Ethics Committee of SUMS (IR.SUMS.MED.REC.1399.337).

### Statistical analysis

The qualitative features were presented as numbers and percentages and the quantitative data were presented as mean (±SD). The non-survivor and survivor groups were compared using independent sample t-test. The time interval from admission date to end of follow-up was regarded censored time if in-hospital death had not occurred*.* The patients’ OS probability was estimated using Kaplan-Meier (KM) curves and the different groups were compared using the non-parametric log-rank test [23].

### Elastic-net regularized Cox-adjusted PH regression

For large sample size data, the regression coefficients can be accurately estimated using traditional maximum likelihood technique [20, 24]. In most medical studies, however, the sample size is not always large enough to estimate reliable and unique coefficients. In such situations, using a regularized version of the likelihood function (i.e. the log partial likelihood function plus a penalty term) can generate reliable results [19, 24]. Ridge and LASSO regressions are two different types of regularization methods that shrink the regression coefficient estimates towards zero to obtain reliable estimates [24, 25]. Unlike ridge regression that will always generate a prognostic model involving all the candidate features, LASSO regression performs feature selection as well. Therefore, LASSO regression results in a sparse model, i.e. a model that involves only a small subset of the candidate features [25]. The elastic-net regularized regression is a convex combination of the ridge and LASSO algorithms [24, 25] and its log partial likelihood function (i.e. *ℓ*_elastic − net_) can be formulated as follows:

1 $${\ell}_{\mathrm{elastic}-\mathrm{net}}=\ell +\mathrm{penalty}$$

where

2 $$\mathrm{penalty}=\uplambda \left(\upalpha \times \mathrm{LASSO}\ \mathrm{penalty}+\left(1-\upalpha \right)\times \mathrm{ridge}\ \mathrm{penalty}\right)$$

Here, *ℓ* is a non-regularized log partial likelihood function, while α and λ are tuning parameters which are data-dependent and some a priori values cannot be attributed to them. The ridge (α=0) and LASSO (α=1) regressions are specific cases of elastic-net regression [24, 25]. More details can be found in Appendix.

The major challenge is to determine these tuning parameters for which the cross-validated likelihood function of the model is maximum. The five-fold cross-validation (CV) approach was used in this study. To perform CV, the original dataset was randomly divided into five equal parts or folds. First, one fold was reserved and a separate model was trained on all the other folds. Then, the trained model was tested on the reserved fold and the partial likelihood deviance was calculated. After repeating this process and utilizing all the five folds as the test sets, the average of the five computed partial likelihood deviances was called the ‘CV error’ [25].

When the sample size is not large enough, instead of the traditional Cox regression, an alternative regularized regression can be used. In the current study, an elastic-net regularized Cox PH regression was employed to model time to in-hospital death in the re-infected COVID-19 patients. Similar to LASSO, the elastic-net algorithm performs feature selection by setting some regression coefficient estimates to zero. The features selected by the elastic-net algorithm were then entered into a standard non-regularized Cox PH regression to specify a baseline for comparison during model development. The backward elimination approach was used to reduce the number of features in the baseline model and to obtain a parsimonious one [24, 25]. In addition, the supremum test was used to check the PH assumption. Finally, we determined the optimal cut-off values of continuous variables using receiver operating characteristic (ROC) curve analysis. The analyses were performed using the “*glmnet*” and “*survival*” packages in the R statistical software (version: 3.6.3) and “PROC PHREG” in SAS statistical software (version 9.2). The MedCalc software (version: 8.0.0.0) was also used to draw the ROC curve for continuous variables, as well as the area under the curve (AUC), 95% CI and p-value calculation.

## Results

The analyses were restricted to 283 patients re-infected with COVID-19 (male: 60%). Out of this number, 178 patients (63%) had underlying diseases (hypertension (28%), kidney diseases (14%), cardiovascular diseases (11%), diabetes mellitus (10%), and others (37%)). The statistics also demonstrated that about 70% of the patients used steroids including dexamethasone, hydrocortisone, and methylprednisolone as adjuvant therapy. With the mean (±SD) age of 52.2 (17.6) years, the empirical in-hospital mortality rate was 9.5%. The descriptive results also indicated that about 81.5% of the deaths occurred during the first 7 days after admission. In addition, the results showed that the in-hospital death rate among the intubated re-infected patients was 83.3%. More details of the baseline demographic characteristics and clinical and laboratory test findings are presented in Tables 1 and 2.

**Table 1:** Comparing the demographic and triage characteristics of the re-infected COVID-19 patients using the non-parametric log-rank test analysis

		Non-survivors	Survivors	Log-rank test
Features		No. (%)^a^	No. (%)^a^	*P*-value
Type of patient transfer	EMS	17 (6.0)	47 (16.6)	**<0.001**
	Not-EMS	10 (3.5)	209 (73.9)	
Age at admission	≤50 years	4 (1.4)	137 (48.4)	**<0.001**
	>50 years	23 (8.1)	119 (42.0)	
Gender	Women	9 (3.2)	105 (37.1)	0.640
	Men	18 (6.4)	151 (53.4)	
SpO_2_ (%)	≤85	21 (7.4)	53 (18.7)	**<0.001**
	>85	6 (2.1)	203 (71.7)	
PR (beats/min)	<60	0 (0.0)	7 (2.5)	**0.025**
	60-119	19 (6.7)	225 (79.5)	
	≥120	8 (2.8)	24 (8.5)	
RR (breaths/min)	≤20	15 (5.3)	189 (66.8)	**0.022**
	>20	12 (4.2)	67 (23.7)	
Temperature (°C)	<37.4	18 (6.4)	208 (73.5)	**0.054**
	≥37.4	9 (3.2)	48 (17.0)	
Triage level	1	15 (5.3)	27 (9.5)	**<0.001**
	2	11 (3.9)	125 (44.2)	
	3	1 (0.4)	104 (36.7)	
Intubation	No	22 (7.8)	255 (90.1)	**<0.001**
	Yes	5 (1.8)	1 (0.4)	

*Note:* The bold numbers indicate the statistically significant factors (*p*-value≤0.05)

*Abbreviations*: *EMS* emergency medical services, *No*. number, *PR* pulse rate, *RR* respiratory rate, *SpO*_*2*_ saturation of peripheral oxygen

^a^*The percentages (%) are calculated across the whole sample of 283* re-infected COVID-19 patients

**Table 2:** Comparing the baseline laboratory test values of the re-infected COVID-19 patients (non-survivors vs. survivors) using independent sample t-test

	Non-survivors	Survivors	Independent sample t-test
Features	Mean (±SD)	Mean (±SD)	*P*-value
DBP (mm Hg)	72.1 (17.4)	80.3 (13.8)	**0.005**
SBP (mm Hg)	126.1 (22.9)	127.7 (19.6)	0.682
Calcium (mg/dL)	8.6 (0.7)	8.8 (0.5)	**0.029**
Potassium (mEq/L)	5.2 (1.0)	4.5 (0.6)	**<0.001**
Sodium (mEq/L)	141.3 (8.5)	140.4 (4.5)	0.380
BUN (mg/dL)	45.3 (27.5)	20.1 (12.8)	**<0.001**
ESR (mm/h)	47.5 (19.8)	46.0 (19.4)	0.711
ALKPH (U/L)	236.3 (70.7)	207.0 (94.5)	0.120
SGPT (U/L)	75.3 (60.2)	57.0 (37.3)	**0.024**
SGOT (U/L)	86.4 (78.8)	52.9 (27.8)	**<0.001**
Phosphore (mg/dL)	3.9 (1.2)	3.4 (0.7)	**0.006**
Albumin (mg/dL)	3.9 (0.4)	4.1 (0.5)	**0.004**
PLT (×10^9^ cells/L)	221.7 (128.9)	267.0 (1.2)	0.055
HCT (%)	40.9 (8.4)	42.3 (5.3)	0.218
Hemoglobin (g/dL)	13.1 (3.0)	13.9 (2.1)	0.083
Creatinine (mg/dL)	2.4 (1.9)	1.3 (0.7)	**<0.001**
WBC count (×10^9^ cells/L)	13.3 (6.3)	8.4 (3.8)	**<0.001**
PT (seconds)	15.8 (2.4)	14.9 (1.7)	**0.014**
PTT (seconds)	43.9 (0.5)	40.1 (9.7)	0.072
T-protein (mg/dL)	6.8 (0.5)	7.0 (0.6)	0.184
Ferritin (ng/mL)	1026.9 (843.7)	891.6 (444.3)	0.178
CPK (mg/dL)	236.4 (234.8)	198.8 (185.4)	0.330
LDH (U/L)	1244.4 (1069.9)	705.9 (229.0)	**<0.001**
D-dimer (ng/mL)	2257.0 (1338.8)	1908.3 (1054.5)	0.113
Magnesium (mg/dL)	2.7 (0.5)	2.5 (0.4)	**0.001**
CRP (mg/L)	23.4 (14.6)	24.2 (16.9)	0.819

*Note*: The bold numbers indicate the statistically significant factors (*p*-value≤0.05)

*Abbreviations*: *ALKPH* alkaline phosphatase, *BUN* blood urea nitrogen, *CPK* creatine phosphokinase, *CRP* C-reactive protein, *DBP* diastolic blood pressure, *ESR* erythrocyte sedimentation rate, *HCT* hematocrit, *LDH* lactate dehydrogenase, *PT* prothrombin time, *PTT* partial thromboplastin time, *PLT* blood platelet, *SBP* systolic blood pressure, *SGPT* serum glutamic pyruvic transaminase, *SGOT* serum glutamic oxaloacetic transaminase, *WBC* white blood cell

Regarding drug treatment (steroids and antibiotics), the majority of the re-infected patients received dexamethasone (*n*=177, 62.5%) followed by lopinavir/ritonavir, branded as Kaletra (*n*=114, 40.3%), azithromycin (*n*=80, 28.3%), remdesivir (*n*=38, 13.4%), chloroquine (*n*=28, 9.9%), hydrocortisone (*n*=27, 9.5%), methylprednisolone (*n*=19, 6.7%), and favipiravir (*n*=7, 2.5%).

### Non-parametric analyses (KM plots and log-rank tests)

**Fig. 1:**
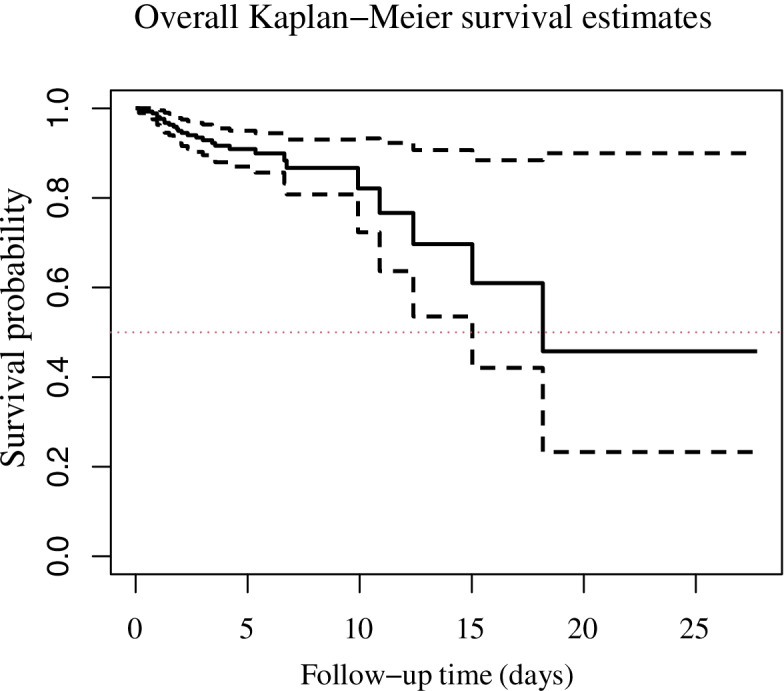
The overall non-parametric Kaplan-Meier survival estimates for the re-infected COVID-19 patients (solid line) and their corresponding 95% CI (dashed lines) (the total analysis time at risk and under observation was equal to 1250 days)

**Fig. 2:**
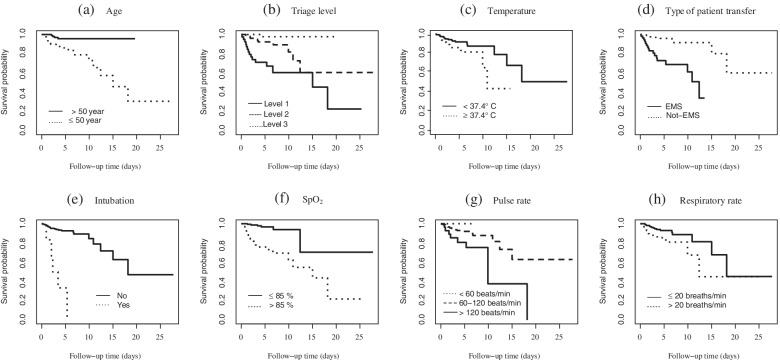
The overall non-parametric Kaplan-Meier survival estimates for the re-infected COVID-19 patients by: **a** age; **b** triage levels; **c** temperature; **d** type of patient transfer; **e** intubation; **f** SpO_2_; **g** pulse rate; **h** respiratory rate

The non-parametric KM plots for the survival probabilities are given in Figs. 1 and 2. The curves detail the time to in-hospital death in the current study. The x-axis represents the elapsed time (in days) from the admission date and the y-axis stands for the survival probabilities. The median of survival time was 18.2 (range: 0.05-27.70) days. As Fig. 1 demonstrates (the dashed lines represent 95% CI), 12.5% of the re-infected COVID-19 patients experienced in-hospital death by the end of the seventh day and 35.3% of them died from that point until the end of the 21-day COVID-19 data collection period.

Based on the non-parametric log-rank test, significant associations with OS were found for the following variables: age (*P*=0.001), type of patient transfer (*P*<0.001), temperature (*P*=0.053), SpO_2_ (*P*<0.001), pulse rate (*P*=0.024), respiratory rate (*P*=0.022), intubation (*P*<0.001), and triage level (*P*<0.0001). Moreover, there was no significant difference in OS by gender (*P*=0.638) (Fig. 2).

### Results of elastic-net regularized Cox-adjusted PH regression

**Fig. 3:**
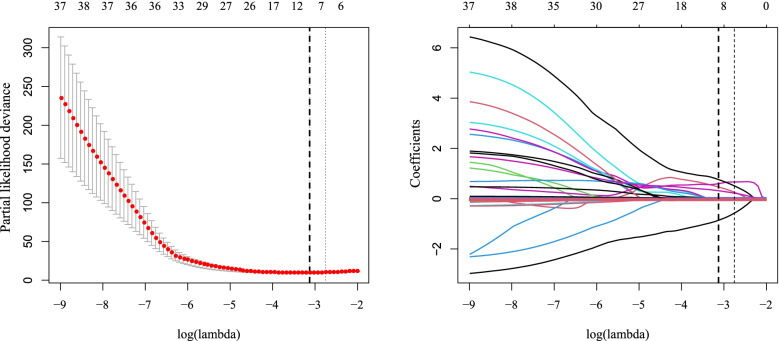
Left: The partial likelihood deviance of five-fold cross validation including lower and upper standard deviations (SDs) as a function of log (lambda) for the dataset of the re-infected COVID-19 patients. The dashed and dotted vertical lines demonstrate the lambda values with a minimal deviance (log λ=-3.2226) and the largest lambda value within one SD of the minimal deviance (log λ=-2.8506), respectively. Right: The elastic-net regularized coefficients on the dataset of the re-infected COVID-19 patients are shown as a function of log (lambda)

The elastic-net regularized Cox-adjusted PH model was trained using a combination of optimized λ values for the ridge (α=0) and LASSO (α=1) regressions. The values of the tuning parameters α and λ were optimized by averaging five repetitions of five-fold CV to minimize the partial likelihood deviance error (α_optimal_=0.9 and λ_optimal_=0.03985) (Fig. 3).

The elastic-net regularized Cox PH model retained 8 out of 35 candidate features of death. The estimated shrunken coefficients for all the retained features are summarized in Table 3. The model parameters may be interpreted in the same way as non-regularized regression parameters whereby lower values show a smaller magnitude of effect. Using the elastic-net regularized regression, the highest magnitude effects belonged to the patients who were transferred to EMDs by EMS (coefficient=0.9145), followed by patients with the SpO_2_ of ≤85% (coefficient=0.8145), intubated patients (coefficient=0.5699), and cases with triage level 1 (coefficient=0.5067). The features selected by the elastic-net regularized regression were then entered into the non-regularized Cox-adjusted PH model to specify a baseline for comparison during model approximation. The stepwise backward elimination method was used to convert the baseline regression model into a parsimonious one.

**Table 3:** The selected features of time to in-hospital death or discharge and the regularized elastic-net coefficients in the re-infected COVID-19 patients (α_optimal_=0.9 and λ_optimal_= 0.03985)

Features	Coefficient ^a^
Type of patient transfer (by EMS)	0.9145
SpO_2_ (≤85%)	0.8145
Intubation (yes)	0.5699
Triage level (level 1 vs. others)	0.5067
Creatinine (mg/dL)	0.3385
WBC count (×10^9^ cells/L)	0.0098
BUN (mg/dL)	0.0082
LDH (U/L)	0.0003

*Abbreviations*: *BUN* blood urea nitrogen, *EMS* Emergency Medical Services, *LDH* lactate dehydrogenase, *SpO*_*2*_ saturation of peripheral oxygen, *WBC* white blood cell

^a^ Estimated coefficients using regularized elastic-net analysis sorted by magnitude from highest to lowest

The results of the elastic-net regularized Cox regression as well as the hazard ratio (HR) (95% CI) of in-hospital death are shown in Table 4. The dataset of the re-infected COVID-19 patients did not show any violation of the PH assumption based on the supremum test results (all the p-values were >0.05). Hence, it was possible to use the analysis of the elastic-net regularized Cox-adjusted regression (Table 4). The coefficients estimated by the model can also be interpreted as the average value of the effect of each feature on the OS rate over time.

**Table 4:** The hazard ratios (95% CIs) for time to in-hospital death in the re-infected COVID-19 patients using multiple regularized elastic-net Cox-adjusted PH regression

Features		HR (95% CI)	***P***-value	PH assumption test*
Type of patient transfer	Not-EMS	Reference	-	-
	EMS	3.90 (1.63-9.48)	**0.002**	0.782
SpO_2_ (%)	>85	Reference	-	-
	≤85	8.10 (2.97-22.00)	**<0.001**	0.132
WBC count (×10^9^ cells/L) for one unit increase		1.10 (1.03-1.15)	**<0.001**	0.895
Creatinine (mg/dL) for one unit increase		1.85 (1.48-2.30)	**0.003**	0.332

*Note*: The significant *p*-values (<0.05) are highlighted in bold

*Abbreviations CI* confidence interval, *dof* degree of freedom, *EMS* Emergency Medical Services, *HR* hazard ratio, *SpO*_*2*_ saturation of peripheral oxygen, *WBC* white blood cell

*The *p*-value for testing the proportional hazards (PH) assumption based on the supremum test

**Fig. 4:**
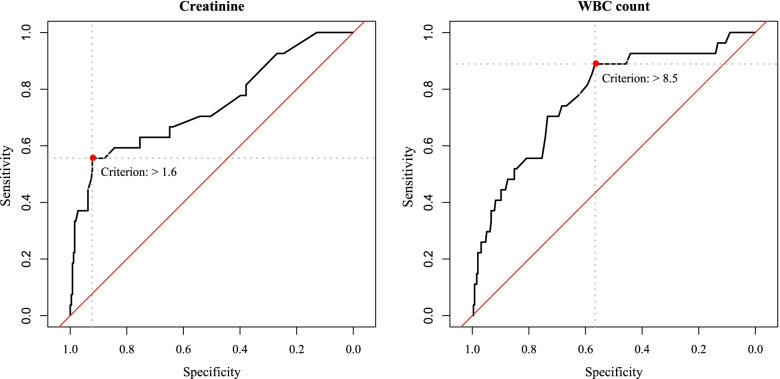
Receiver operating characteristic (ROC) curves for prediction of overall survival in the re-infected COVID-19 patients for creatinine (left: AUC=0.742 (95% CI: 0.687-0.792, *P*<0.001)) and WBC count (right: AUC=0.772 (95% CI: 0.719–0.820, *P*<0.001))

The elastic-net ML analysis indicated that transfer to EMDs by EMS (HR=3.90, 95% CI: 1.63-9.48), SpO_2_ of ≤85% (HR=8.10, 95% CI: 2.97-22.00), increased serum creatinine (HR=1.85, 95% CI: 1.48-2.30), and increased WBC count (HR=1.10, 95% CI: 1.03-1.15) were associated with higher mortality rates in the re-infected COVID-19 patients. In addition, the ROC curve analysis suggested that the cut-off values of 8.5 (×10^9^ cells/L) for WBC count and 1.6 mg/dL for serum creatinine were the best to distinguish between patient’s OS (WBC count: AUC=0.772 (95% CI: 0.719–0.820, *P*<0.001) and creatinine: AUC=0.742 (95% CI: 0.687-0.792, *P*<0.001) (Fig. 4).

## Discussion

The review of literature showed that no research has been done so far on the predictive determinants of overall survival among re-infected COVID-19 patients. Only the systematic review conducted by SeyedAlinaghi et al. was a comprehensive study which assessed the risk of COVID-19 re-infection [6]. They found thirty-one eligible studies of which eight studies described the patients who recovered from COVID-19 re-infection and only one study reported death among them. However, the majority of the published works (26 studies) did not present any extra information about the patients’ status (i.e. death or discharge) [6].

The underlying diseases, clinical conditions, use of glucocorticoids, and secondary bacterial infection were identified as the independent risk factors of COVID-19 re-infection [6, 26, 27]. In addition, although re-infection is possible, it should be noted that the re-infection or reactivation diagnosed in some patients might in fact be a false negative at the time of discharge or not meeting the discharge criteria completely. On the other hand, three main reasons including short-lived, ineffective, and strain-specific immune responses may lead to a positive PCR test result [28, 29].

Recent studies have reported that some patients who had recovered from COVID-19 had a positive PCR test result for the second time [5, 8, 30–36]. For instance, it was stated in a report that 116 patients in South Korea who had recovered from COVID-19 had positive PCR test results again [33]. In addition, most previously published works which described patients with COVID-19 re-infection were in the format of case reports [5, 8, 30–32, 34–36] and no studies evaluated the OS and its related predictors among these patients.

Regularization algorithms such as elastic-net and LASSO can be used to perform feature selection and to improve the prediction accuracy by shrinking the coefficients towards zero [24]. In this study, two ML algorithms (elastic-net regularized Cox-adjusted PH model and backward stepwise elimination) were applied to the dataset of re-infected COVID-19 patients to predict the OS and the associated factors among them. The current study is unique in that it incorporates all regularized algorithms under the elastic-net umbrella. These algorithms created two models. One of them maximized parsimony and the other optimized the predictive power. The elastic-net Cox-adjusted PH regression kept 8 out of 35 candidate features of time to discharge or in-hospital death. The strongest predictors (i.e. the features with the highest magnitude of the estimated coefficients) included the type of patient transfer (using the EMS or not), SpO_2_, intubation, and triage level (level 1 vs. others). The backward elimination method further reduced the regularized model to retain four features: type of patient transfer, SpO_2_, WBC count, and serum creatinine.

Since no similar studies were found about the survival of re-infected COVID-19 patients, the results of this study were compared with those of the studies related to survival and the related risk factors in patients with COVID-19. The results of the current research showed that the empirical in-hospital mortality rate was 9.5%. Furthermore, the OS rates for days 7, 14, and 21 were obtained as 87.5, 78.3, and 52.2%, respectively, in the re-infected COVID-19 inpatients. These rates have been reported differently for COVID-19 patients in other studies [12, 15, 37]. For example, Murillo-Zamora and Hernandez-Suarez found that 7-, 15-, 21- and 30-day OS rates were respectively 72.2, 47.6, 35.0, and 23.9% which were lower than the results obtained in the current study [37]. In another study by Sousa et al., the 24-day OS rate in 2070 patients with COVID-19 was calculated as 87.7% [15].

Regarding the laboratory findings at the time of admission, it was found that increased serum creatinine (more than 1.6 mg/dL) and increased WBC count (more than 8.5 (×10^9^ cells/L)) were associated with a higher mortality rate in re-infected COVID-19 patients. As compared with the surviving re-infected COVID-19 patients, the levels of creatinine were independent predictors of abnormal kidney function at the time of admission in the non-surviving re-infected COVID-19 patients. The higher in-hospital mortality rate was related to the higher concentration levels of creatinine (>1.6 mg/dL) in the patients, suggesting a worse renal function at the time of hospital admission. This finding is in line with previous studies which revealed that the concentration levels of creatinine were significantly higher among the COVID-19 patients who died [38–40].

Moradi et al. assessed the risk of one-month mortality from COVID-19 since the time of admission. They found that increased NLR and increased WBC count were associated with a higher one-month death rate. Moreover, although hypoxemia (SpO_2_ <90%) increased the one-month mortality rate, this association was not significant [18]. After adjustment for confounders, the results of the present study demonstrated that higher SpO_2_ levels (greater than 85%) after oxygen supplementation were associated with reduced mortality. In fact, profound hypoxemia (SpO_2_≤85%) could have a harmful effect on the OS of re-infected COVID-19 patients, increasing the risk of mortality eight-fold. The findings of the present study were consistent with previous studies in which profound hypoxemia was associated with a higher in-hospital death rate [41, 42].

Another survey by Yan et al. applied an ML-based algorithm to predict OS among 404 patients with severe COVID-19. They reported three biomarkers including lymphocyte, lactic dehydrogenase (LDH), and high-sensitivity C-reactive protein (hs-CRP) as the survival predictors with the accuracy of more than 90%. In particular, it was revealed that high levels of LDH might have an independent harmful effect on the OS rate [43].

We could not compare our results with other studies because we did not find any studies reporting transfer by EMS as an OS predictive factor. However, it could be said that the patients who were transferred to EMDs by EMS had a more severe status, increasing their mortality rate almost four-fold.

This study had several limitations which should be mentioned. We could not find any similar study in the literature to compare our findings with. Therefore, we had to compare our results with studies which used general COVID-19 datasets for their analyses. The impossibility of examining the risk factors associated with re-infection as well as the difficulty of confirming the diagnosis of COVID-19 re-infection were two other limitations of the present study. Another limitation of this study was that it was conducted during the peak period of infection especially when the virus had an active transmission chain among the populations. Hence, our findings may vary in non-pandemic conditions.

## Conclusion

On the basis of the results it was concluded that transfer by the EMS, profound hypoxemia (SpO_2_≤85%), increased serum creatinine (more than 1.6 mg/dL), and increased WBC count (more than 8.5 (×10^9^ cells/L)) reduced the OS of re-infected COVID-19 patients. Finally, we recommend that future machine-learning studies should further explore these relationships and the associated factors in these patients for a better prediction of OS.

## Supplementary Information

**Additional file 1: Appendix**

## Data Availability

The datasets used and analyzed during the current study are available from the corresponding authors on reasonable request.

## Abbreviations

ALKPH: Alkaline phosphatase
BUN: Blood urea nitrogen
COVID-19: Severe acute respiratory syndrome coronavirus 2 (SARS-CoV-2) disease 2019
CV: Cross validation
CI: Confidence interval
CPK: Creatine phosphokinase
CRP: C-reactive protein
dof: Degree of freedom
DBP: Diastolic blood pressure
EMD: Emergency Medicine Department
EMS: Emergency Medical Services
ESR: Erythrocyte sedimentation rate
HR: Hazard ratio
hs-CRP: High-sensitivity C-reactive protein
HIS: Health Information System
HCT: Hematocrit
Ig: Immunoglobulin
ICU: Intensive care unit
KM: Kaplan-Meier
LDH: Lactic dehydrogenase
LASSO: Least absolute shrinkage and selection operator
LDH: Lactate dehydrogenase
ML: Machine learning
NLR: Neutrophil-to-lymphocyte ratio
OS: Overall survival
OEPCF: Outcome events per candidate feature
PH: Proportional hazards
PR: Pulse rate
P: *P*-value
PT: Prothrombin time
PTT: Partial thromboplastin time
PLT: Blood platelet
RT-PCR: Reverse transcription polymerase chain reaction
RR: Respiratory rate
SpO_2_: Saturation of peripheral oxygen
SBP: Systolic blood pressure
SGPT: Serum glutamic pyruvic transaminase
SGOT: Serum glutamic oxaloacetic transaminase
SUMS: Shiraz University of Medical Sciences
SD: Standard deviation
WBC: White blood cell
WHO: World Health Organization
