# Supplementary material for: Predictive determinants of overall survival among re-infected COVID-19 patients using the elastic-net regularized Cox proportional hazards model: a machine-learning algorithm
Source: BMC Public Health. 2022 Jan 5;22:10. doi: 10.1186/s12889-021-12383-3 (PMC8727465; doi:10.1186/s12889-021-12383-3)
Supplement: Supplementary file 1 — Additional file 1: Appendix [file 12889_2021_12383_MOESM1_ESM.docx]

**Appendix**

**Elastic-net regularized Cox-adjusted PH regression**

Suppose we have data of the form $\left( \text{T}_{\text{j}}\text{, }\text{x}_{\text{j}}\text{, }\text{δ}_{\text{j}} \right)\text{, j=1, 2}\text{, …,}\text{ n}$ where $\text{T}_{\text{j}}$, is the observed time of right-censoring if $\text{δ}_{\text{j}}$=0 or event time if $\text{δ}_{\text{j}}$=1. As in ordinary multiple regression, $\text{x}_{\text{j}}$ is a vector of potential covariates $\text{(}\text{x}_{\text{j1}}\text{, }\text{x}_{\text{j2}}\text{, …,}\text{ }\text{x}_{\text{jK}}\text{)}$. We further let $\text{t}_{\text{1}}\text{<}\text{t}_{\text{2}}\text{<…<}\text{t}_{\text{p}}$ be the increasing sequence of unique event times, and *i*(*j*) denotes the index of the observation failing at time $\text{t}_{\text{j}}$.

The standard non-regularized Cox PH regression assumes a semi-parametric form for the hazard

$$\text{h}_{\text{j}}\left( \text{t} \right)\text{=}\text{h}_{\text{0}}\left( \text{t} \right)\text{×}\exp\left\{ \text{x}_{\text{j}}^{\boldsymbol{'}}\text{γ} \right\}\text{=}\text{h}_{\text{0}}\left( \text{t} \right)\text{×exp\{}\sum_{\text{i=1}}^{\text{K}} \text{γ}_{\text{i}}\text{x}_{\text{ji}}\text{\} }\text{ }\text{ }\text{(1)}$$

where $\text{h}_{\text{j}}\left( \text{t} \right)$ is the hazard function for patient j at time t, $\text{h}_{\text{0}}\left( \text{t} \right)$ is a baseline hazard function, and $\text{γ}\text{=(}\text{γ}_{\text{1}}\text{, }\text{γ}_{\text{2}}\text{, …, }\text{γ}_{\text{K}}\text{)}$ is a fixed, length K vector. Inference is then made through the partial likelihood function

$$\text{L}\left( \text{γ} \right)\text{=}\prod_{\text{j=1}}^{\text{p}} \text{\{}\text{exp(} \text{x}_{\text{i(}\text{j}\text{)}}^{\boldsymbol{'}}\text{γ}\text{)}\text{/}\sum_{\text{i}\text{∈}\text{R}_{\text{j}}} \exp\text{(}\text{x}_{\text{j}}^{\boldsymbol{'}}\text{γ}\text{)}\text{\}}\text{ (2).}$$

Here, $R_{j}$ is the set of indices, *i*, with $\text{T}_{\text{i}}\text{≥}\text{t}_{\text{j}}$ (patients at risk at the time of $\text{t}_{\text{j}}$). By maximizing the log partial likelihood function, one can estimate $\text{γ}$ (1-3)**.**

For classical situations, with many more patients than candidate features, the non-regularized Cox-adjusted PH regression performs well. However, for small sample size data, using traditional Cox regression can be misleading if the number of the candidate features is relatively large and the number of the outcome events per candidate feature is small (4). In such cases, using regularized Cox PH regression through machine-learning (ML) algorithms is the better option. These regularized models solve this problem by adding a penalty term to the log partial likelihood function. The regularization techniques such as LASSO and elastic-net are popularly applied for feature selection in the ML domain. The elastic-net algorithm is a hybrid of LASSO and ridge regressions (3-5). The elastic-net regularized Cox partial log-likelihood function (i.e. $\mathcal{l}_{\text{elastic-net}}$) is defined as

$$\mathcal{l}_{\text{elastic-net}}\left( \text{γ} \right)\text{=}\text{ log(}\text{L}\left( \text{γ} \right)\text{)+}\text{P}_{\text{α, λ}}\left( \text{γ} \right)\text{=}\sum_{j=1}^{p} (\text{x}_{\text{i(j)}}^{\boldsymbol{'}}\text{γ}-\log\left( \sum_{\text{i}\text{∈}\text{R}_{\text{j}}} \exp\text{(}\text{x}_{\text{j}}^{\boldsymbol{'}}\text{γ}\text{)} \right))+\text{P}_{\text{α, λ}}\left( \text{γ} \right) (3)$$

$\text{ }$where

$\text{P}_{\text{α, λ}}\left( \text{γ} \right)\text{=}\sum_{\text{i=1}}^{\text{K}} \text{λ}(\text{α}\left| \text{γ}_{\text{i}} \right|\text{+}\frac{\text{1}}{\text{2}}\text{(1-α)}\text{γ}_{\text{j}}^{\text{2}}) (4)$ is the penalty term that regularized the estimates. Here, $\text{λ≥0}$ and *α*∈[0, 1] (3, 5, 6). The elastic-net simplifies to simple LASSO regularization when α=1 and to the ridge regularization when α=0 (5). Furthermore, when λ=0, the penalty term is eliminated and the objective function reduces to the usual partial log-likelihood function. As λ increases, however, more and more model parameters shrink to 0. The trick is to specify the optimal values of the regularization parameters λ and α.

In practice, for any pre-specified α value, the optimal value of λ is determined according to the common fitness measures such as cross-validation on the given dataset (3). Cross-validation partitions the analysis dataset into several disjoint folds. Start with reserving one fold. Estimate the parameters with the remaining folds. The criterion such as Bayesian information criterion (BIC), deviance or Harrell's concordance index, is applied to the fold that is reserved. Repeat the process by reserving one fold at a time. The criterion is then averaged across the folds. In this study, we considered the following sequence of α parameter: *α*=0, 0.1, 0.2, 0.3, 0.4, 0.5, 0.6, 0.7, 0.8, 0.9, and 1 (*α*=0 ridge and *α*=1 LASSO regularization). For each *α*, we used the cross-validated partial log-likelihood deviance to select the appropriate regularization parameter λ so the smallest value of deviance is preferred (3, 5). The cyclical coordinate descent algorithm was utilized for maximizing the partial log-likelihood with the elastic-net penalty. The “*glmnet”* R package version 3.0-2 was used for training the elastic-net regularized Cox PH model (3).

**References**:

1. Christensen E. Multivariate survival analysis using Cox's regression model. Hepatology. 1987;7(6):1346-58. doi: <https://doi.org/10.1002/hep.1840070628>.

2. David G, Kleinbaum K. Survival analysis: a self-learning text: Springer-Verlag New York; 2016.

3. Friedman J, Hastie T, Tibshirani R. glmnet: Lasso and elastic-net regularized generalized linear models 2009 [cited 1 4]. 1-24]. Available from: <https://cran.r-project.org/web/packages/glmnet/index.html>.

4. Peduzzi P, Concato J, Kemper E, Holford TR, Feinstein AR. A simulation study of the number of events per variable in logistic regression analysis. Journal of clinical epidemiology. 1996;49(12):1373-9. doi: <https://doi.org/10.1016/S0895-4356(96)00236-3>.

5. Zou H, Hastie T. Regularization and variable selection via the elastic net. Journal of the royal statistical society: series B (statistical methodology). 2005;67(2):301-20. doi: <https://doi.org/10.1111/j.1467-9868.2005.00503.x>.

6. Simon N, Friedman J, Hastie T, Tibshirani R. Regularization paths for Cox’s proportional hazards model via coordinate descent. Journal of statistical software. 2011;39(5):1. doi: <https://doi.org/10.18637/jss.v039.i05>.
